# Targeting type 2 immunity and the future of food allergy treatment

**DOI:** 10.1084/jem.20221104

**Published:** 2023-03-03

**Authors:** M. Cecilia Berin

**Affiliations:** 1 Northwestern University Feinberg School of Medicine, Chicago, IL, USA

## Abstract

IgE-mediated food allergy affects 6–8% of the population in the United States. Type 2 immune responses are central to the pathogenesis of food allergy, but type 2 CD4^+^ T cell responses have been found to be heterogeneous in food allergy suggesting a division of labor between Tfh13 and peTH2 cells in promotion of IgE class switching, modulation of intestinal barrier function, and regulation of mast cell expansion. Oral immunotherapy for the treatment of food allergy incompletely targets subsets of type 2 immunity in a transient manner, but new therapeutics targeting different levels of type 2 immunity are in current or planned trials for food allergy. These new treatments and the basis for their use are the focus of this review.

## Teaser

There is growing appreciation of the heterogeneity of type 2 immunity in food allergy and how type 2 cytokines from distinct cell types contribute to pathogenesis. The landscape of clinical trials in food allergy is being transformed with type 2–targeting therapeutics.

## Introduction

Food allergy refers to reproducible immune-mediated adverse reactions to foods (Boyce et al., 2010) and includes a range of disorders including IgE-mediated food allergy, food protein-induced enterocolitis syndrome, and eosinophilic esophagitis. This review will focus on IgE-mediated food allergy, which affects 6–8% of the US population (Sampath et al., 2021). Although sensitization to foods often occurs in the first months of life before introduction of the food into the diet, adult-onset IgE-mediated food allergy is not uncommon (Gupta et al., 2019; Warren et al., 2020). The threshold of reactivity varies, with some individuals reacting to low milligram quantities that can be present as trace contaminants during food preparation or processing. Severity also varies, with symptoms ranging from mild itching to vomiting and diarrhea to life-threatening respiratory compromise. There is only one FDA-approved treatment for peanut allergy (Palforzia, a standardized peanut powder used for oral desensitization). However, there are several clinical trials in various stages of completion testing novel approaches to immunotherapy, in the presence or absence of immunomodulatory biologicals (Ramsey and Berin, 2021). In this review, the immune phenotype of food allergy and impact of successful oral immunotherapy (OIT) treatment on the food allergen-specific immune response will be discussed, with a view to the next generation of food allergy OIT.

## Immune profile of food allergy

### Immunoglobulin profile

IgE-mediated food allergy is by definition associated with the presence of specific IgE antibodies against food proteins. However, the presence of food allergen-specific IgE, referred to as “sensitization,” is not itself diagnostic for food allergy. Levels of specific IgE are related to the probability of having food allergy, and for a number of foods 95% predictive values of specific IgE have been identified (Celik-Bilgili et al., 2005; Sampson, 2001). The relationship of IgE to probability of clinical reactivity varies by population and by age. For some foods, measurement of IgE against defined allergens (“components”) provides a greater predictive value than IgE against the whole food extract. This has been shown for peanut, hazelnut, and soy (Hemmings et al., 2022; Keet et al., 2021). This is in part due to the cross-reactivity of IgE to proteins homologous to birch in birch-allergic individuals that may not trigger allergic reactions beyond the oral mucosa when ingested (Werfel et al., 2015). Even greater specificity can be obtained by examining epitope-specific IgE. This has been demonstrated using linear epitopes from peanut, egg, and milk (Suárez-Fariñas et al., 2019; Suprun et al., 2018; Suprun et al., 2022b; Suprun et al., 2020). Not only clinical reactivity, but threshold of reactivity has been predicted using such an approach (Suprun et al., 2022a). This suggests that not all IgE epitopes are clinically meaningful, perhaps due to gastrointestinal processing and lack of availability of such epitopes for IgE binding after digestion and absorption. Phage display techniques have suggested an important role for conformational epitopes in peanut allergy that are not represented by linear epitope arrays (Chen et al., 2016). Beyond IgE, levels of other isotypes of food-specific antibodies such as IgG, IgG4, or IgA are not predictive of clinical reactivity at steady state.

### B cell profile

Allergen-specific B cells have been identified in peripheral blood by labeling with fluorescent food allergen multimers (Hoh et al., 2015; Patil et al., 2015). Sorting of such cells has identified allergen-specific IgG, IgA, and IgM secreting B cells, but IgE secreting B cells are exceedingly rare (Patil et al., 2015). Croote et al. (2018) sorted 973 B cells enriched for surface IgE expression from peanut allergic individuals, and of these 89 were confirmed by sequencing to be true IgE-producing B cells (Croote et al., 2018). These were primarily plasmablasts, in contrast to IgG clones that were memory or naive B cells. These cells were marked with high levels of CD23 and were Class II high, indicating a more immature phenotype of plasmablast. In addition, low levels of Syk suggested a reduced survival ability. IgE antibodies cloned from B cells expressing common gene rearrangements across different individuals showed a high affinity cross-reactive binding to Ara h 2 and Ara h 3, two clinically important peanut allergens. Jiménez-Saiz et al. (2019) attempted to identify IgE^+^ memory B cells from humans through a first selection of B cells followed by sequential gating out of IgD, IgM, IgA, and IgG positive cells (Jiménez-Saiz et al., 2019). Single-sorted cells then underwent nested PCR to confirm IgE expression. No IgE expressing cells were identified from 10 peanut allergic individuals. Thus, true IgE^+^ cells can be identified as plasmablasts but not memory B cells. While food allergy is often lifelong, the basis of that is not well understood. IgE^+^ plasma cells are generally thought to be short-lived, but under conditions of chronic antigen exposure, long-lived plasma cells have been shown to populate the bone marrow of mice (Asrat et al., 2020). It remains to be tested in humans if long-lived IgE^+^ plasma cells specific for food allergens are present in the bone marrow. It has been shown that IgE-producing clones with plasma cell markers are present and highly enriched in the human upper gastrointestinal tract (stomach and duodenum) of peanut allergic individuals (Hoh et al., 2020), suggesting alternative anatomical locations for a reservoir of long-lived plasma cells.

Studies in mice have provided a mechanistic explanation for the lack of memory IgE B cells. It has been shown that high-affinity IgE must undergo sequential class-switch from IgG to IgE (Erazo et al., 2007; Xiong et al., 2012). The capacity for IgE memory was shown to be contained within IgG^+^ memory B cells, and those cells required STAT6-dependent CD4^+^ T cell help and antigen exposure in order to continually class-switch to IgE and maintain IgE levels over time (He et al., 2017; Jimenez-Saiz et al., 2017). The IgE found in the human gastrointestinal tract is clonally related to IgA also found in the same site, demonstrating potential for local class switch from IgA1 to IgE (Hoh et al., 2020). Together, these data indicate that T cells are required not only for initiation of IgE production but also for maintenance of IgE production by continual renewal from memory B cells of other isotypes.

### T cell profile in food allergy

It has been appreciated for many years that food allergy was associated with an allergen-specific type 2 cytokine profile, defined by production of IL-4, IL-5, and IL-13 from CD4^+^ T cells. Initial studies grew lines from patient-derived PBMCs, and demonstrated type 2 cytokine production from the lines (de Jong et al., 1996; Higgins et al., 1995). CFSE-labeling of peanut-reactive T cells indicated an abundance of IL-4, IL-5, and IL-13 producing T cells from peanut allergic donors compared to donors who had outgrown their peanut allergy (Turcanu et al., 2003). Growing lines or allowing cells to proliferate in culture can alter the phenotype of responder cells. Studies using activation marker-based approaches to identification of allergen-specific CD4^+^ T cells that examined the cytokine phenotype after as little as 4–6 h of stimulation ex vivo showed a robust Th2 skewing of T cells from peanut or egg allergic individuals (Berin et al., 2018; Chiang et al., 2018; Prussin et al., 2009; Ruiter et al., 2020; Wambre et al., 2017). Controls who were sensitized but not reactive, or healthy controls, showed a much reduced frequency of antigen-specific T cells and a lack of type 2 skewing (Chiang et al., 2018; Prussin et al., 2009). In addition to IL-4, IL-5, and IL-13, IL-9 has been identified as an important CD4^+^ T cell cytokine associated with the peanut-specific immune response (Brough et al., 2014; Chiang et al., 2018). Type 2 T cells were shown to be highly differentiated memory T cells, expressing multiple type 2 cytokines (IL-4, IL-5, IL-13, IL-9) and lacking expression of CD27 (Chiang et al., 2018; Wambre et al., 2017). One subset of highly differentiated type 2 cells co-express CRTH2, CD49d, and CD161 and have been termed Th2A cells (Wambre et al., 2017). Others have referred to the cells as pathogenic effector Th2 cells, and variably described CRTH2 expression on the cells (Chiang et al., 2018; Monian et al., 2022; Prussin et al., 2009; Ruiter et al., 2020). For simplicity, we will refer to highly differentiated multi-cytokine producing type 2 CD4^+^ T cells as peTh2. Type 2 cells express CCR4, while CCR6 expression on peanut- or egg-specific T cells negatively correlates with IL-4 and IgE production (Berin et al., 2022).

### T cell regulation of IgE

The cytokine IL-4 drives IgE class-switch, and this provides an explanation of how type 2 T cells contribute to the pathophysiology of food allergy. Allergen-specific type 2 T cells correlate with circulating levels of specific IgE (Berin et al., 2022). However, much of the focus has historically been on Th2 cells, while T follicular helper (Tfh) cells are required to initiate class-switch due to their location in the B cell follicle where they can interact with naive B cells. Gowthaman et al. (2019) identified a novel population of Tfh cells that co-expressed IL-4 and IL-13, and also expressed high levels of IL-5 and low levels of IL-21 (Gowthaman et al., 2019). These cells co-expressed the transcription factors GATA3 and BCL6. These cells, termed Tfh13 cells, were necessary for induction of high-affinity IgE that could support the generation of anaphylaxis in mice. Furthermore, analysis of T cells from peanut allergic individuals demonstrated that CXCR5^+^ T cells co-expressing IL-4 and IL-13 could be detected in peanut allergic but not control individuals. Dolence et al. (2018) used a mouse model of peanut allergy initiated by airway sensitization to demonstrate that Tfh cells were necessary for peanut-induced anaphylaxis (Dolence et al., 2018). Thus, a Tfh source of type 2 cytokines is necessary for peanut allergy in mice.

Bruton et al. (2021) studied T cell regulation of IgE production during a recall response to peanut allergen (Bruton et al., 2021). They found that blocking IL-4/IL-13 signaling inhibited the production of allergen-induced specific IgE in a human PBMC model system ex vivo and a mouse model in vivo. However, the subset of T cell important for the production of IgE during a memory response is not yet identified.

### Impact of type 2 T cells beyond IgE

The finding that Tfh cell subsets are critical for the induction of anaphylaxis in mice leads to the question of the role of the peTh2 cell. These cells are found in the periphery, and in eosinophilic disorders are found to be highly enriched in the gastrointestinal tract (Mitson-Salazar et al., 2016; Morgan et al., 2021). It is not known if they are enriched in the gastrointestinal tract in IgE-mediated food allergy, but that is likely based on their presence in the periphery. There is evidence for IgE class switch in the gastrointestinal mucosa in humans (Hoh et al., 2020), and based on mouse models (He et al., 2017; Jimenez-Saiz et al., 2017), this would require type 2 cell help. peTh2 cells may provide such a tissue B cell help function. Gastrointestinal mast cells play a critical role in food allergy in mice (Osterfeld et al., 2010), and the burden of gastrointestinal mast cells relates to systemic reactivity to foods (Ahrens et al., 2012). IL-9 is a key cytokine promoting mast cell expansion, and has been shown to be derived from peTh2 cells in food allergy (Chiang et al., 2018). IL-9 gene expression is elevated in the intestine of individuals with food allergy, although this may also be derived from a unique subset of IL-9-producing mast cells termed MC9 cells (Chen et al., 2015).

**Figure 1.**
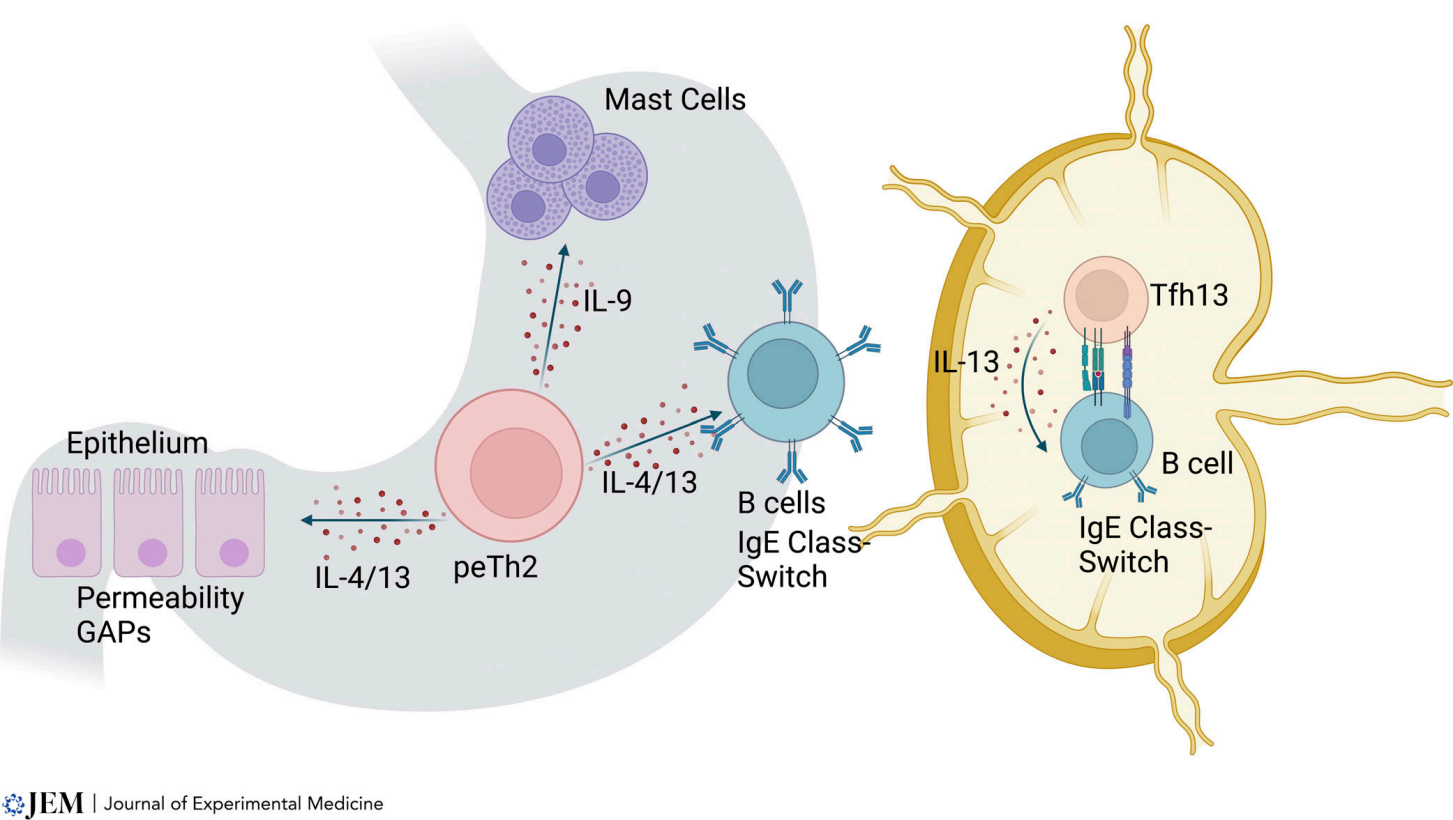
**Type 2 cell subsets play diverse roles in the pathogenesis of food allergy.** Tfh13 and peTh2 cells have been identified in food allergy. Tfh13 cells are likely responsible for the initiation of class switch and generation of food-specific IgE. peTh2 cells are found in the periphery, and likely play a tissue helper role through promotion of mast cell expansion, increased epithelial permeability to food antigens, and support of local IgE class switch from memory IgG cells.

Another major contributing factor to food allergy is the regulation of epithelial barrier function and uptake of food antigens (Newberry and Hogan, 2021). Goblet cells and other secretory cells have been shown to be involved in the uptake of dietary antigens through passages that allow rapid penetration of antigen (Noah et al., 2019). These are called goblet cell associated passages, or GAPs. These too are under the regulation of type 2 cytokines, particularly IL-13. In addition to this transcellular route, there is evidence that type 2 cytokines regulate the tight junctions of enterocytes allowing for the paracellular passage of macromolecules (Berin et al., 1999). Fig. 1 highlights the influence of type 2 cytokines in gastrointestinal tissues and lymph nodes in food allergy.

### Tregs and food allergy

In mouse models of food allergy, a key role for regulatory T cells has also been demonstrated (Noval Rivas and Chatila, 2016). Mice with susceptibility to food allergy due to a gain-of-function IL-4R have an abundance not only of conventional Th2 cells but also Tregs producing IL-4 (Noval Rivas et al., 2015). Deletion of IL-4 under the Foxp3 promoter could eliminate susceptibility to food allergy, despite the fact that the mice still had conventional CD4^+^ type 2 T cells. These IL-4 and GATA3 expressing Tregs were also identified in milk allergic individuals. Others have used the Treg-specific activation marker CD137 to study peanut-specific Tregs in humans, and failed to find a difference in frequency of these cells in peanut allergic versus healthy controls (Weissler et al., 2018). Natural resolution of food allergy has been shown to be associated with an expansion of antigen-specific Tregs (Qamar et al., 2015).

### Innate immunity in food allergy

The innate immune system also appears to contribute to the pathogenesis of food allergy. Zhang et al. (2016) found that infants who went on to develop food allergy at 1 yr of life had hyper-inflammatory monocytes that could support Th2 skewing in the absence of IL-2 (Zhang et al., 2016). An altered monocyte phenotype in the context of established food allergy has also been described by Neeland et al. (2018, 2020). It is not clear how monocytes may be contributing to disease as they are not thought to be dominant antigen presenting cells. However, they may be representative of the mononuclear phagocyte system, and changes in monocyte phenotype, i.e., “trained immunity,” may also be present within tissue dendritic cells (DCs) that prime T cells to their effector fate, or in tissue macrophages that set the inflammatory tone of tissues.

Innate cells can also play a key role by either promoting type 2 responses or serving as a source of type 2 cytokines. Innate lymphoid cells type 2 (ILC2) play a key role in mouse models of food allergy by amplifying the production of type 2 cytokines (Lee et al., 2016; Noval Rivas et al., 2016). Innate cytokines that drive type 2 immunity, namely TSLP, IL-33, and IL-25, have also been shown to be necessary for the development of food allergy in mouse models (Han et al., 2018; Khodoun et al., 2018; Lee et al., 2016). The role of ILC2s and type 2–inducing cytokines has not been as well examined in humans due to the difficulty in getting access to relevant tissue specimens such as the gastrointestinal tract.

## Immunotherapy for food allergy

### Oral immunotherapy

The first FDA-approved therapy for the treatment of peanut allergy was recently approved and is a standardized peanut powder given as oral immunotherapy (Palforzia). This follows many years of clinical trials for the treatment of peanut and other food allergies by oral immunotherapy (Burks et al., 2012; Jones et al., 2009; Skripak et al., 2008; Varshney et al., 2009; Varshney et al., 2011). The majority of individuals who start on OIT therapy can become “desensitized,” meaning that they are protected against allergic reactions to that specific food while they are maintained on treatment. However, in clinical trials ∼10–20% of individuals discontinue due to persistent side effects (primarily gastrointestinal side effects) that often resolve on termination of the treatment (Virkud et al., 2017). While the goal of commercial OIT treatment is desensitization, a goal of the field is to get to a state of “remission,” where individuals can stop daily treatment and maintain the level of clinical protection. This is also referred to as “sustained unresponsiveness” (SU). Studies on the maintenance of clinical protection have shown that the longer the period of avoidance, the fewer individuals maintain remission (Chinthrajah et al., 2019a). Younger children, particularly in the first year of life, appear to have a higher rate of remission, but it is not yet clear if there is a window of opportunity to achieve true tolerance in response to OIT (Jones et al., 2022; Vickery et al., 2017).

### Immune response to OIT

The first studies of the immune response to OIT demonstrated that specific IgG4 levels rise over time, IgE levels may initially rise but drop over time, Th2 cytokines measured in recall assays are suppressed over time, and Tregs were noted to increase in some studies (Burks et al., 2012; Jones et al., 2009; Syed et al., 2014; Thyagarajan et al., 2012; Varshney et al., 2011; Vickery et al., 2014). An increase in the mucosal neutralizing antibody isotype IgA has also been identified in saliva and serum (Kulis et al., 2012; Wright et al., 2016). Studies that compared SU to other outcomes demonstrated that a lower IgE level at baseline was associated with development of SU (Vickery et al., 2014). An early rise in IgG4 was associated with SU, while at later timepoints IgG4 did not discriminate between those with or without SU (Burks et al., 2012). Basophil activation test was also an early predictor of the development of SU (Patil et al., 2019; Tsai et al., 2020). Neutralization of allergen by IgG4 and IgA likely contribute to protective mechanisms of OIT, and IgG antibodies may also contribute by activating phosphatases downstream of FcγRII (Burton et al., 2014; Santos et al., 2015). The use of monoclonal IgG antibodies to provide protection against peanut reactivity may be a future therapeutic approach, as has been tested for birch and cat allergy (de Blay et al., 2022; Gevaert et al., 2022).

Studies examining T cells at the single-cell level, either by flow cytometry or single cell RNAseq found that there was a reduction in frequency of type 2 T cells in response to oral immunotherapy (Bajzik et al., 2022; Berin et al., 2022; Monian et al., 2022; Wambre et al., 2017; Wisniewski et al., 2015). Studies specifically examining Th2A cells found a decreased frequency after OIT treatment (Bajzik et al., 2022; Wambre et al., 2017). Monian et al. (2022) used a combination of TCR sequencing and transcriptional profiling to track changes in phenotype of peanut-specific T cells over time, using cells selected as CD154^+^ or CD137^+^ after stimulation with peanut extract (Monian et al., 2022). CD154^+^ cells enrich for activated antigen-specific T effector cells, while CD137 selects for antigen-specific regulatory CD4^+^ T cells (Bacher et al., 2016). Cells of both Th2 and Th1 phenotype were suppressed by OIT, but suppression of cells specifically belonging to a Th2A-like subset were associated with a positive clinical outcome (Monian et al., 2022). Interestingly, there was no impact of OIT on Tfh2 cells or Th2-like Tregs. In the Consortium for Food Allergy Research, we observed by flow cytometry that egg-specific type 2 cells were suppressed by egg OIT, and were significantly higher at baseline in those who experienced treatment failure (Berin et al., 2022). Those of the top tertile by frequency of egg-specific IL-4^+^ CD4^+^ T cells experienced a treatment failure rate at twice the level of those in the low or mid tertiles. Thus, type 2 immunity appears to be instrumental in treatment success or failure.

### Adjunct therapies for food allergy OIT

A number of adjunct therapies are in the pipeline for use with OIT, with the goal of decreasing side effects or enhancing remission. Omalizumab, or anti-IgE therapy, has been tested together with single-food or multi-food OIT in a number of small clinical trials (Bégin et al., 2014; MacGinnitie et al., 2017; Nadeau et al., 2011; Schneider et al., 2013; Wood et al., 2016). Omalizumab increases the safety of OIT by reducing reactions to the treatment itself, but does not appear to enhance remission (Wood et al., 2016). A current trial run by the Consortium for Food Allergy Research is testing the impact of omalizumab alone or with multi-food OIT (NCT03881696). A theoretical benefit of omalizumab is its antigen non-specific effect, which would provide much broader protection than single-food OIT. Omalizumab could potentially be used on its own to prevent allergic reactions (Sampson et al., 2011), used with OIT to make OIT safer (Wood et al., 2016), or potentially be used together with dietary food introduction (Fiocchi et al., 2019). The latter two would involve eventual tapering of omalizumab, if found to be safe. A newer higher-affinity version of anti-IgE, ligelizumab, is currently being tested as a stand-alone therapy for peanut allergy (NCT04984876).

Removal of IgE does not remove the IgE-producing plasma cells. If research from mouse models is correct, continual T cell help by IL-4/IL-13 producing T cells is necessary for maintenance of IgE (Bruton et al., 2021; He et al., 2017; Jimenez-Saiz et al., 2017). Dupilumab, which binds and neutralizes the IL-4Ra subunit of both IL-4 and IL-13 receptors, recently completed trials as both a monotherapy (NCT03793608) and together with peanut OIT (NCT03682770). As a monotherapy, it was reported that 2 of 24 participants treated with dupilumab were able to tolerate a 444 mg cumulative peanut challenge after 24 wk of treatment, and this was accompanied by an ∼50% reduction in peanut-specific IgE (NCT03793608). It would be expected that these modest effects would be amplified by addition of OIT where presence of antigen in the absence of IL-4/IL-13 signaling may be more tolerogenic. Abrocitinib, an inhibitor of JAK1 that is part of the signaling complex downstream of a number of cytokine receptors including IL-4, IL-13, IL-9, and TSLP, is also in a phase I trial for the treatment of food allergy (NCT05069831). This is in part a mechanistic study, examining the impact of abrocitinib on basophil activation tests in place of oral food challenges, and on peanut-specific type 2 and Treg cell responses. In unpublished pre-clinical studies, abrocitinib was shown to suppress basophil activation and Th2 cytokine production from peanut allergic subjects in vitro. As with dupilumab, it may be more effective to target JAK signaling in association with OIT than as a monotherapy. If successful, targeting signaling downstream of IL-4 and IL-13 will starve the B cells of the help that they need to class switch and maintain IgE levels. Abrocitinib would also target IL-9 that expands mast cells, and TSLP, a driver of Th2 responses.

Another therapeutic approach is to interfere with type 2 immune generation. Dendritic cells imprint naive T cells to Th2 cells in part though the expression of the co-stimulatory molecule OX40L. Neutralization of OX40L can prevent type 2 skewing and sensitization in mice (Blázquez and Berin, 2008; Chu et al., 2012). An anti-OX40L antibody amlitelimab has been shown to have efficacy in atopic dermatitis (Lé and Torres, 2022). Targeting OX40 on T cells has also shown considerable promise as a treatment for atopic dermatitis (Guttman-Yassky et al., 2022), and suppressed type 2 inflammation in the skin (Guttman-Yassky et al., 2019).

**Figure 2.**
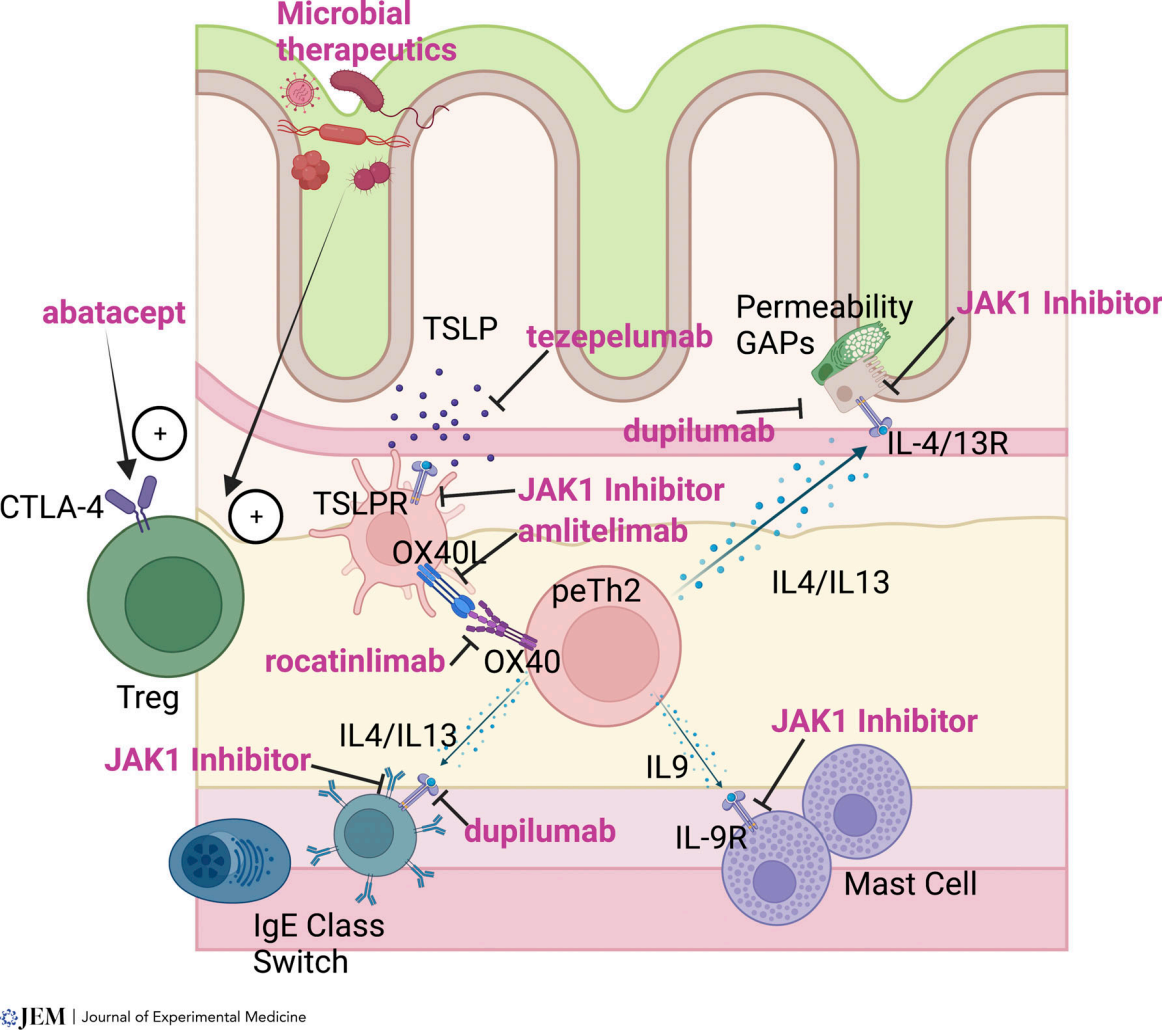
**Therapeutics targeting type 2 immunity in food allergy.** The schematic illustrates type 2 immunity at the center of food allergy pathogenesis through actions on B cells, mast cells, and epithelium. Shown in pink text are inhibitors currently in trials or proposed as therapies for food allergy. Dupilumab and JAK1 inhibitors would act to prevent the action of type 2 cytokines, while tezepelumb, amlitelimab, and rocatinlimab prevent the formation of type 2 immunity. Microbial therapeutics and abatacept promote Tregs that suppress type 2 immunity.

TSLP is one of a trio of cytokines that drive type 2 immunity (also including IL-33 and IL-25), and these could form targets to eliminate the source of type 2-driving activity. TSLP upregulates OX40L on DCs to promote Th2 skewing, as well as activating basophils. Neutralization of TSLP with tezepelumab has been used together with cat allergen immunotherapy, with greater clinical efficacy than immunotherapy alone (Corren et al., 2022). Gene expression measured from nasal brushings demonstrated reduced expression of a gene module that was enriched for mast cell and type 2 cytokine genes. It would be of interest to test this target in the context of food allergy. Neutralization of the Th2-inducing cytokine IL-33 with etokimab was previously tested as a monotherapy in peanut allergy (Chinthrajah et al., 2019b), with some promising results, but it was not tested together with OIT and has not been continued. In a mouse model of food allergy, neutralization of all Th2-driving cytokines IL-25, IL-33, and TSLP was necessary to suppress symptoms, but this was not performed in the context of OIT (Khodoun et al., 2018). Fig. 2 illustrates the presumed mechanism of action of type 2-targeting therapies in food allergy.

Microbial therapies are also being developed for treatment of food allergy, as stand-alone therapies or combined with OIT. Readers are referred to other publications for a thorough review of the role of the microbiota in food allergy (Rachid et al., 2021), but it has been established that the intestinal microbiota is altered in food allergy, that alterations precede the onset of disease, and that transfer of allergic vs. healthy microbiota to germ-free mice can transfer susceptibility to disease. Approaches to microbial treatments include the use of conventional probiotics together with OIT (Tang et al., 2015), fecal microbial transplants (FMT; NCT02960074), and the development of multi-microbe cocktails that have shown efficacy in mouse models (NCT03936998). Probiotics with OIT failed to show greater efficacy than OIT alone, although did protect against gastrointestinal side effects (Loke et al., 2022). FMT and multi-microbe cocktails have not yet reported results from their clinical trials. The microbiota are thought to function by normalizing or enhancing the function of gastrointestinal Tregs to suppress Type 2 responses. One additional approach to promoting Tregs in food allergy is the use of the CTLA-4 agonist abatacept. CTLA-4 has been shown to play a key role in food tolerance (Krempski et al., 2022; van Wijk et al., 2005), and abatacept is being used as an adjunct therapy to peanut OIT (NCT04872218). Table 1 summarizes the trials that are currently in progress or unpublished that are discussed in this manuscript.

**Table 1.** Ongoing studies of novel therapeutics in food allergy

Identifier	Therapeutic	Target	*n*	Description
NCT03881696	Omalizumab + multi-food OIT	IgE	225	RDBPC study examining omalizumab as a monotherapy, and as an adjunct to multi-food OIT in peanut (+ 2 foods) allergic participants. 1° outcome is the percentage of subjects who pass a 600 mg DBPCPC at 16–20 wk.
NCT04984876	Ligelizumab	IgE	486	52-wk phase 3 study RDBPC trial. 1° outcome is the percentage of subjects who pass a 600 mg DBPCPC challenge at wk 12.
NCT03793608	Dupilumab	IL-4/IL-13 receptors	25	24 wk open label study. 1° outcome is the percentage of participants passing a DPBCPC of 444 mg or greater.
NCT03682770	Dupilumab + AR101	IL-4/IL-13 receptors	149	RDBPC study to evaluate the efficacy of dupilumab as an adjunct therapy to AR101 (peanut OIT). 1° outcome is the percentage of participants who pass a 2044 mg DBPCPC.
NCT05069831	Abrocitinib	Cytokines signaling through JAK1	40	A double-blind randomized study testing 100 and 200 mg of abrocitinib for 16 wk. 1° outcome is change in peanut-induced basophil activation from baseline to 16 wk.
NCT02960074	FMT	Microbiota	15	1 yr two-arm study of FMT (*n* = 10, phase I) or antibiotics + FMT (*n* = 5, phase II). 1° outcome is FMT-related adverse events grade 2 or higher. 2° endpoints include change in threshold of peanut reactivity during a DBPCPC.
NCT03936998	VE416 + peanut OIT	Microbiota	60	RDBPC study to evaluate the impact of a microbial cocktail (VE416) with or without vancomycin on peanut OIT. 1° outcome (phase 1) is number of participants with treatment-related adverse events; 1° outcome (phase 2) is maximum tolerated dose of peanut during a DBPCPC.
NCT04872218	Abatacept + peanut OIT	CTLA-4	14	RDBPC study comparing 24 wk of abatacept vs. placebo with OIT. 1° outcome is peanut-specific IgE at wk 24. 2° endpoints include threshold dose, and sustained unresponsiveness at 36 wk.

RDBPC, randomized double-blind placebo-controlled; DBPCPC, double-blind placebo-controlled peanut challenge.

## Future directions

A few years ago, there was intense effort to understand what the reservoir of IgE memory was, with the result that we understand that the IgE memory is held within the pool of IgG^+^ memory cells as precursors. Targeting type 2 immunity should interrupt the maintenance of allergen-specific IgE, but would likely need to be a lifelong treatment unless the reservoir of type 2 memory is also targeted. Type 2 responses, including frequency of Th2A cells, are suppressed by OIT, yet they rebound when treatment stops. Understanding the factors necessary for generation of type 2 effector cells from central memory cells, and more importantly for maintenance of type 2 cell memory (particularly within affected tissues such as the gastrointestinal tract), is essential to developing long-lasting therapeutics. The development of personalized approaches to treatment is an unmet need. For example, the finding that high type 2 cell frequency at baseline is associated with OIT treatment failure suggests a method to identify individuals for whom dupilumab or JAK1 inhibition may be a necessary adjunct to OIT. We understand that there is heterogeneity in the type 2 response (peTh2 vs*.* Tfh13), and that type 2 subsets are differentially suppressed by OIT (Monian et al., 2022), but do not yet understand the implications for therapy. Although we are beginning to understand phenotypic variability in food allergy (for example, high vs. low threshold of reactivity), we do not yet have established endotypes in food allergy. The clinical trials currently in progress, if appropriately paired with mechanistic studies, will advance the field of personalized medicine in food allergy by relating high-resolution immune phenotype to treatment response.

## References

[bib1] Ahrens, R., H. Osterfeld, D. Wu, C.Y. Chen, M. Arumugam, K. Groschwitz, R. Strait, Y.H. Wang, F.D. Finkelman, and S.P. Hogan. 2012. Intestinal mast cell levels control severity of oral antigen-induced anaphylaxis in mice. Am. J. Pathol. 180:1535–1546. 10.1016/j.ajpath.2011.12.036 22322300 PMC3354589

[bib2] Asrat, S., N. Kaur, X. Liu, L.H. Ben, D. Kajimura, A.J. Murphy, M.A. Sleeman, A. Limnander, and J.M. Orengo. 2020. Chronic allergen exposure drives accumulation of long-lived IgE plasma cells in the bone marrow, giving rise to serological memory. Sci. Immunol. 5:5. 10.1126/sciimmunol.aav8402 31924685

[bib3] Bacher, P., F. Heinrich, U. Stervbo, M. Nienen, M. Vahldieck, C. Iwert, K. Vogt, J. Kollet, N. Babel, B. Sawitzki, . 2016. Regulatory T cell specificity directs tolerance versus allergy against aeroantigens in humans. Cell. 167:1067–1078.e16. 10.1016/j.cell.2016.09.050 27773482

[bib4] Bajzik, V., H.A. DeBerg, N. Garabatos, B.J. Rust, K.K. Obrien, Q.A. Nguyen, C. O’Rourke, A. Smith, A.H. Walker, C. Quinn, . 2022. Oral desensitization therapy for peanut allergy induces dynamic changes in peanut-specific immune responses. Allergy. 77:2534–2548. 10.1111/all.15276 35266148 PMC9356972

[bib5] Bégin, P., T. Dominguez, S.P. Wilson, L. Bacal, A. Mehrotra, B. Kausch, A. Trela, M. Tavassoli, E. Hoyte, G. O’Riordan, . 2014. Phase 1 results of safety and tolerability in a rush oral immunotherapy protocol to multiple foods using Omalizumab. Allergy Asthma Clin. Immunol. 10:7. 10.1186/1710-1492-10-7 24576338 PMC3936817

[bib6] Berin, M.C., C. Agashe, A.W. Burks, D. Chiang, W.F. Davidson, P. Dawson, A. Grishin, A.K. Henning, S.M. Jones, E.H. Kim, . 2022. Allergen-specific T cells and clinical features of food allergy: Lessons from CoFAR immunotherapy cohorts. J. Allergy Clin. Immunol. 149:1373–1382.e12. 10.1016/j.jaci.2021.09.029 34653515 PMC8995337

[bib7] Berin, M.C., A. Grishin, M. Masilamani, D.Y.M. Leung, S.H. Sicherer, S.M. Jones, A.W. Burks, A.K. Henning, P. Dawson, J. Grabowska, . 2018. Egg-specific IgE and basophil activation but not egg-specific T-cell counts correlate with phenotypes of clinical egg allergy. J. Allergy Clin. Immunol. 142:149–158.e8. 10.1016/j.jaci.2018.01.044 29518422 PMC6282170

[bib8] Berin, M.C., P.C. Yang, L. Ciok, S. Waserman, and M.H. Perdue. 1999. Role for IL-4 in macromolecular transport across human intestinal epithelium. Am. J. Physiol. 276:C1046–C1052. 10.1152/ajpcell.1999.276.5.C1046 10329951

[bib9] Blázquez, A.B., and M.C. Berin. 2008. Gastrointestinal dendritic cells promote Th2 skewing via OX40L. J. Immunol. 180:4441–4450. 10.4049/jimmunol.180.7.4441 18354165

[bib10] Boyce, J.A., A. Assa’ad, A.W. Burks, S.M. Jones, H.A. Sampson, R.A. Wood, M. Plaut, S.F. Cooper, M.J. Fenton, and S.H. Arshad, . 2010. Guidelines for the diagnosis and management of food allergy in the United States: Summary of the NIAID-sponsored expert panel report. J. Allergy Clin. Immunol. 126:1105–1118. 10.1016/j.jaci.2010.10.008 21134568 PMC4241958

[bib11] Brough, H.A., D.J. Cousins, A. Munteanu, Y.F. Wong, A. Sudra, K. Makinson, A.C. Stephens, M. Arno, L. Ciortuz, G. Lack. . 2014. IL-9 is a key component of memory TH cell peanut-specific responses from children with peanut allergy. J. Allergy Clin. Immunol. 134:1329–1338.e10. 10.1016/j.jaci.2014.06.032 25112699

[bib12] Bruton, K., P. Spill, S. Vohra, O. Baribeau, S. Manzoor, S. Gadkar, M. Davidson, T.D. Walker, J.F.E. Koenig, Y. Ellenbogen, . 2021. Interrupting reactivation of immunologic memory diverts the allergic response and prevents anaphylaxis. J. Allergy Clin. Immunol. 147:1381–1392. 10.1016/j.jaci.2020.11.042 33338539

[bib13] Burks, A.W., S.M. Jones, R.A. Wood, D.M. Fleischer, S.H. Sicherer, R.W. Lindblad, D. Stablein, A.K. Henning, B.P. Vickery, and A.H. Liu, . 2012. Oral immunotherapy for treatment of egg allergy in children. N. Engl. J. Med. 367:233–243. 10.1056/NEJMoa1200435 22808958 PMC3424505

[bib14] Burton, O.T., S.L. Logsdon, J.S. Zhou, J. Medina-Tamayo, A. Abdel-Gadir, M. Noval Rivas, K.J. Koleoglou, T.A. Chatila, L.C. Schneider, R. Rachid, . 2014. Oral immunotherapy induces IgG antibodies that act through FcγRIIb to suppress IgE-mediated hypersensitivity. J. Allergy Clin. Immunol. 134:1310–1317.e6. 10.1016/j.jaci.2014.05.042 25042981 PMC4261076

[bib15] Celik-Bilgili, S., A. Mehl, A. Verstege, U. Staden, M. Nocon, K. Beyer, and B. Niggemann. 2005. The predictive value of specific immunoglobulin E levels in serum for the outcome of oral food challenges. Clin. Exp. Allergy. 35:268–273. 10.1111/j.1365-2222.2005.02150.x 15784102

[bib16] Chen, C.Y., J.B. Lee, B. Liu, S. Ohta, P.Y. Wang, A.V. Kartashov, L. Mugge, J.P. Abonia, A. Barski, K. Izuhara, . 2015. Induction of interleukin-9-producing mucosal mast cells promotes susceptibility to IgE-mediated experimental food allergy. Immunity. 43:788–802. 10.1016/j.immuni.2015.08.020 26410628 PMC4618257

[bib17] Chen, X., S.S. Negi, S. Liao, V. Gao, W. Braun, and S.C. Dreskin. 2016. Conformational IgE epitopes of peanut allergens Ara h 2 and Ara h 6. Clin. Exp. Allergy. 46:1120–1128. 10.1111/cea.12764 27238146 PMC4963300

[bib18] Chiang, D., X. Chen, S.M. Jones, R.A. Wood, S.H. Sicherer, A.W. Burks, D.Y.M. Leung, C. Agashe, A. Grishin, P. Dawson, . 2018. Single-cell profiling of peanut-responsive T cells in patients with peanut allergy reveals heterogeneous effector T_H_2 subsets. J. Allergy Clin. Immunol. 141:2107–2120. 10.1016/j.jaci.2017.11.060 29408715 PMC5994177

[bib19] Chinthrajah, R.S., N. Purington, S. Andorf, A. Long, K.L. O’Laughlin, S.C. Lyu, M. Manohar, S.D. Boyd, R. Tibshirani, H. Maecker, . 2019a. Sustained outcomes in oral immunotherapy for peanut allergy (POISED study): A large, randomised, double-blind, placebo-controlled, phase 2 study. Lancet. 394:1437–1449. 10.1016/S0140-6736(19)31793-3 31522849 PMC6903389

[bib20] Chinthrajah, S., S. Cao, C. Liu, S.C. Lyu, S.B. Sindher, A. Long, V. Sampath, D. Petroni, M. Londei, and K.C. Nadeau. 2019b. Phase 2a randomized, placebo-controlled study of anti-IL-33 in peanut allergy. JCI Insight. 4:4. 10.1172/jci.insight.131347 PMC6948865 31723064

[bib21] Chu, D.K., A. Llop-Guevara, T.D. Walker, K. Flader, S. Goncharova, J.E. Boudreau, C.L. Moore, T.S. In, S. Waserman, A.J. Coyle, . 2012. IL-33, but not thymic stromal lymphopoietin or IL-25, is central to mite and peanut allergic sensitization. J. Allergy Clin. Immunol. 131:187–200.e1-8. 10.1016/j.jaci.2012.08.002 23006545

[bib22] Corren, J., D. Larson, M.C. Altman, R.M. Segnitz, P.C. Avila, P.A. Greenberger, F. Baroody, M.H. Moss, H. Nelson, A.J. Burbank, . 2022. Effects of combination treatment with tezepelumab and allergen immunotherapy on nasal responses to allergen: A randomized controlled trial. J. Allergy Clin. Immunol. 151:192–201. 10.1016/j.jaci.2022.08.029 36223848 PMC12205947

[bib23] Croote, D., S. Darmanis, K.C. Nadeau, and S.R. Quake. 2018. High-affinity allergen-specific human antibodies cloned from single IgE B cell transcriptomes. Science. 362:1306–1309. 10.1126/science.aau2599 30545888

[bib24] de Blay, F.J., A. Gherasim, N. Domis, P. Meier, F. Shawki, C.Q. Wang, J.M. Orengo, M. DeVeaux, D. Ramesh, J.J. Jalbert, . 2022. REGN1908/1909 prevented cat allergen-induced early asthmatic responses in an environmental exposure unit. J. Allergy Clin. Immunol. 150:1437–1446. 10.1016/j.jaci.2022.06.025 35934082

[bib25] de Jong, E.C., S. Spanhaak, B.P. Martens, M.L. Kapsenberg, A.H. Penninks, and E.A. Wierenga. 1996. Food allergen (peanut)-specific TH2 clones generated from the peripheral blood of a patient with peanut allergy. J. Allergy Clin. Immunol. 98:73–81. 10.1016/S0091-6749(96)70228-2 8765820

[bib26] Dolence, J.J., T. Kobayashi, K. Iijima, J. Krempski, L.Y. Drake, A.L. Dent, and H. Kita. 2018. Airway exposure initiates peanut allergy by involving the IL-1 pathway and T follicular helper cells in mice. J. Allergy Clin. Immunol. 142:1144–1158.e8. 10.1016/j.jaci.2017.11.020 29247716 PMC6002896

[bib27] Erazo, A., N. Kutchukhidze, M. Leung, A.P. Christ, J.F. Urban Jr, M.A. Curotto de Lafaille, and J.J. Lafaille. 2007. Unique maturation program of the IgE response in vivo. Immunity. 26:191–203. 10.1016/j.immuni.2006.12.006 17292640 PMC1892589

[bib28] Fiocchi, A., M.C. Artesani, C. Riccardi, M. Mennini, V. Pecora, V. Fierro, V. Calandrelli, L. Dahdah, and R.L. Valluzzi. 2019. Impact of omalizumab on food allergy in patients treated for asthma: A real-life study. J Allergy Clin Immunol Pract. 7:1901–1909 e1905. 10.1016/j.jaip.2019.01.023 30797778

[bib29] Gevaert, P., J. De Craemer, N. De Ruyck, S. Rottey, J. de Hoon, P.W. Hellings, B. Volckaert, K. Lesneuck, J.M. Orengo, A. Atanasio, . 2022. Novel antibody cocktail targeting Bet v 1 rapidly and sustainably treats birch allergy symptoms in a phase 1 study. J. Allergy Clin. Immunol. 149:189–199. 10.1016/j.jaci.2021.05.039 34126156

[bib30] Gowthaman, U., J.S. Chen, B. Zhang, W.F. Flynn, Y. Lu, W. Song, J. Joseph, J.A. Gertie, L. Xu, M.A. Collet, . 2019. Identification of a T follicular helper cell subset that drives anaphylactic IgE. Science. 365:883. 10.1126/science.aaw6433 PMC6901029 31371561

[bib31] Gupta, R.S., C.M. Warren, B.M. Smith, J. Jiang, J.A. Blumenstock, M.M. Davis, R.P. Schleimer, and K.C. Nadeau. 2019. Prevalence and severity of food allergies among US adults. JAMA Netw. Open. 2:e185630. 10.1001/jamanetworkopen.2018.5630 30646188 PMC6324316

[bib32] Guttman-Yassky, E., A.B. Pavel, L. Zhou, Y.D. Estrada, N. Zhang, H. Xu, X. Peng, H.C. Wen, P. Govas, G. Gudi, . 2019. GBR 830, an anti-OX40, improves skin gene signatures and clinical scores in patients with atopic dermatitis. J. Allergy Clin. Immunol. 144:482–493.e7. 10.1016/j.jaci.2018.11.053 30738171

[bib33] Guttman-Yassky, E., E.L. Simpson, K. Reich, K. Kabashima, K. Igawa, T. Suzuki, H. Mano, T. Matsui, E. Esfandiari, and M. Furue. 2022. An anti-OX40 antibody to treat moderate-to-severe atopic dermatitis: A multicentre, double-blind, placebo-controlled phase 2b study. Lancet. 401:204–214. 10.1016/S0140-6736(22)02037-2 36509097

[bib34] Han, H., F. Roan, L.K. Johnston, D.E. Smith, P.J. Bryce, and S.F. Ziegler. 2018. IL-33 promotes gastrointestinal allergy in a TSLP-independent manner. Mucosal Immunol. 11:394–403. 10.1038/mi.2017.61 28656964 PMC5745299

[bib35] He, J.S., S. Subramaniam, V. Narang, K. Srinivasan, S.P. Saunders, D. Carbajo, T. Wen-Shan, N. Hidayah Hamadee, J. Lum, A. Lee, . 2017. IgG1 memory B cells keep the memory of IgE responses. Nat. Commun. 8:641. 10.1038/s41467-017-00723-0 28935935 PMC5608722

[bib36] Hemmings, O., U. Niazi, M. Kwok, S. Radulovic, G. Du Toit, G. Lack, and A.F. Santos. 2022. Combining allergen components improves the accuracy of peanut allergy diagnosis. J. Allergy Clin. Immunol. Pract. 10:189–199. 10.1016/j.jaip.2021.08.029 34492400

[bib37] Higgins, J.A., J.R. Lamb, R.A. Lake, and R.E. O’Hehir. 1995. Polyclonal and clonal analysis of human CD4+ T-lymphocyte responses to nut extracts. Immunology. 84:91–97. 7534268 PMC1415172

[bib38] Hoh, R.A., S.A. Joshi, J.Y. Lee, B.A. Martin, S. Varma, S. Kwok, S.C.A. Nielsen, P. Nejad, E. Haraguchi, P.S. Dixit, . 2020. Origins and clonal convergence of gastrointestinal IgE^+^ B cells in human peanut allergy. Sci. Immunol. 5:5. 10.1126/sciimmunol.aay4209 PMC7691169 32139586

[bib39] Hoh, R.A., S.A. Joshi, Y. Liu, C. Wang, K.M. Roskin, J.Y. Lee, T. Pham, T.J. Looney, K.J. Jackson, V.P. Dixit, . 2015. Single B-cell deconvolution of peanut-specific antibody responses in allergic patients. J. Allergy Clin. Immunol. 137:157–167. 10.1016/j.jaci.2015.05.029 26152318 PMC4699867

[bib40] Jimenez-Saiz, R., D.K. Chu, T.S. Mandur, T.D. Walker, M.E. Gordon, R. Chaudhary, J. Koenig, S. Saliba, H.J. Galipeau, A. Utley, . 2017. Lifelong memory responses perpetuate humoral TH2 immunity and anaphylaxis in food allergy. J. Allergy Clin. Immunol. 140:1604–1615 e1605. 10.1016/j.jaci.2017.01.018 28216433 PMC6298600

[bib41] Jiménez-Saiz, R., Y. Ellenbogen, K. Bruton, P. Spill, D.D. Sommer, H. Lima, S. Waserman, S.U. Patil, W.G. Shreffler, and M. Jordana. 2019. Human BCR analysis of single-sorted, putative IgE^+^ memory B cells in food allergy. J. Allergy Clin. Immunol. 144:336–339.e6. 10.1016/j.jaci.2019.04.001 30959060 PMC7010227

[bib42] Jones, S.M., E.H. Kim, K.C. Nadeau, A. Nowak-Wegrzyn, R.A. Wood, H.A. Sampson, A.M. Scurlock, S. Chinthrajah, J. Wang, and R.D. Pesek, . . 2022. Efficacy and safety of oral immunotherapy in children aged 1-3 years with peanut allergy (the immune tolerance network IMPACT trial): A randomised placebo-controlled study. Lancet. 399:359–371. 10.1016/S0140-6736(21)02390-4 35065784 PMC9119642

[bib43] Jones, S.M., L. Pons, J.L. Roberts, A.M. Scurlock, T.T. Perry, M. Kulis, W.G. Shreffler, P. Steele, K.A. Henry, M. Adair, . 2009. Clinical efficacy and immune regulation with peanut oral immunotherapy. J. Allergy Clin. Immunol. 124:292–300, 300 e291-297. 10.1016/j.jaci.2009.05.022 19577283 PMC2725434

[bib44] Keet, C., M. Plesa, D. Szelag, W. Shreffler, R. Wood, J. Dunlop, R. Peng, J. Dantzer, R.G. Hamilton, A. Togias, . 2021. Ara h 2-specific IgE is superior to whole peanut extract-based serology or skin prick test for diagnosis of peanut allergy in infancy. J. Allergy Clin. Immunol. 147:977–983.e2. 10.1016/j.jaci.2020.11.034 33483152 PMC8462936

[bib45] Khodoun, M.V., S. Tomar, J.E. Tocker, Y.H. Wang, and F.D. Finkelman. 2018. Prevention of food allergy development and suppression of established food allergy by neutralization of thymic stromal lymphopoietin, IL-25, and IL-33. J. Allergy Clin. Immunol. 141:171–179.e1. 10.1016/j.jaci.2017.02.046 28552763

[bib46] Krempski, J.W., J.K. Lama, K. Iijima, T. Kobayashi, M. Matsunaga, and H. Kita. 2022. A mouse model of the LEAP study reveals a role for CTLA-4 in preventing peanut allergy induced by environmental peanut exposure. J. Allergy Clin. Immunol. 150:425–439.e3. 10.1016/j.jaci.2022.02.024 35288169 PMC9378358

[bib47] Kulis, M., K. Saba, E.H. Kim, J.A. Bird, N. Kamilaris, B.P. Vickery, H. Staats, and A.W. Burks. 2012. Increased peanut-specific IgA levels in saliva correlate with food challenge outcomes after peanut sublingual immunotherapy. J. Allergy Clin. Immunol. 129:1159–1162. 10.1016/j.jaci.2011.11.045 22236732 PMC3763925

[bib48] Lé, A.M., and T. Torres. 2022. OX40-OX40L inhibition for the treatment of atopic dermatitis-focus on rocatinlimab and amlitelimab. Pharmaceutics. 14:14. 10.3390/pharmaceutics14122753 PMC9787630 36559247

[bib49] Lee, J.B., C.Y. Chen, B. Liu, L. Mugge, P. Angkasekwinai, V. Facchinetti, C. Dong, Y.J. Liu, M.E. Rothenberg, S.P. Hogan, . 2016. IL-25 and CD4^+^ TH2 cells enhance type 2 innate lymphoid cell-derived IL-13 production, which promotes IgE-mediated experimental food allergy. J. Allergy Clin. Immunol. 137:1216–1225 e1211. 10.1016/j.jaci.2015.09.019 26560039 PMC4826796

[bib50] Loke, P., F. Orsini, A.C. Lozinsky, M. Gold, M.D. O’Sullivan, P. Quinn, M. Lloyd, S.E. Ashley, S. Pitkin, and C. Axelrad, . 2022. Probiotic peanut oral immunotherapy versus oral immunotherapy and placebo in children with peanut allergy in Australia (PPOIT-003): A multicentre, randomised, phase 2b trial. Lancet Child Adolesc. Health. 6:171–184. 10.1016/S2352-4642(22)00006-2 35123664

[bib51] MacGinnitie, A.J., R. Rachid, H. Gragg, S.V. Little, P. Lakin, A. Cianferoni, J. Heimall, M. Makhija, R. Robison, R.S. Chinthrajah, . 2017. Omalizumab facilitates rapid oral desensitization for peanut allergy. J. Allergy Clin. Immunol. 139:873–881.e8. 10.1016/j.jaci.2016.08.010 27609658 PMC5369605

[bib52] Mitson-Salazar, A., Y. Yin, D.L. Wansley, M. Young, H. Bolan, S. Arceo, N. Ho, C. Koh, J.D. Milner, K.D. Stone, . 2016. Hematopoietic prostaglandin D synthase defines a proeosinophilic pathogenic effector human T_H_2 cell subpopulation with enhanced function. J. Allergy Clin. Immunol. 137:907–918.e9. 10.1016/j.jaci.2015.08.007 26431580

[bib53] Monian, B., A.A. Tu, B. Ruiter, D.M. Morgan, P.M. Petrossian, N.P. Smith, T.M. Gierahn, J.H. Ginder, W.G. Shreffler, and J.C. Love. 2022. Peanut oral immunotherapy differentially suppresses clonally distinct subsets of T helper cells. J. Clin. Invest. 132:132. 10.1172/JCI150634 PMC8759778 34813505

[bib54] Morgan, D.M., B. Ruiter, N.P. Smith, A.A. Tu, B. Monian, B.E. Stone, N. Virk-Hundal, Q. Yuan, W.G. Shreffler, and J.C. Love. 2021. Clonally expanded, GPR15-expressing pathogenic effector T_H_2 cells are associated with eosinophilic esophagitis. Sci. Immunol. 6:6. 10.1126/sciimmunol.abi5586 PMC8686696 34389613

[bib55] Nadeau, K.C., L.C. Schneider, L. Hoyte, I. Borras, and D.T. Umetsu. 2011. Rapid oral desensitization in combination with omalizumab therapy in patients with cow’s milk allergy. J. Allergy Clin. Immunol. 127:1622–1624. 10.1016/j.jaci.2011.04.009 21546071 PMC3396422

[bib56] Neeland, M.R., J.J. Koplin, T.D. Dang, S.C. Dharmage, M.L. Tang, S.L. Prescott, R. Saffery, D.J. Martino, and K.J. Allen. 2018. Early life innate immune signatures of persistent food allergy. J. Allergy Clin. Immunol. 142:857–864.e3. 10.1016/j.jaci.2017.10.024 29154959

[bib57] Neeland, M.R., B. Novakovic, T.D. Dang, K.P. Perrett, J.J. Koplin, and R. Saffery. 2020. Hyper-inflammatory monocyte activation following endotoxin exposure in food allergic infants. Front. Immunol. 11:567981. 10.3389/fimmu.2020.567981 33072108 PMC7541825

[bib58] Newberry, R.D., and S.P. Hogan. 2021. Intestinal epithelial cells in tolerance and allergy to dietary antigens. J. Allergy Clin. Immunol. 147:45–48. 10.1016/j.jaci.2020.10.030 33144143

[bib59] Noah, T.K., K.A. Knoop, K.G. McDonald, J.K. Gustafsson, L. Waggoner, S. Vanoni, M. Batie, K. Arora, A.P. Naren, Y.H. Wang, . 2019. IL-13-induced intestinal secretory epithelial cell antigen passages are required for IgE-mediated food-induced anaphylaxis. J. Allergy Clin. Immunol. 144:1058–1073.e3. 10.1016/j.jaci.2019.04.030 31175877 PMC6779525

[bib60] Noval Rivas, M., O.T. Burton, H.C. Oettgen, and T. Chatila. 2016. IL-4 production by group 2 innate lymphoid cells promotes food allergy by blocking regulatory T-cell function. J. Allergy Clin. Immunol. 138:801–811.e9. 10.1016/j.jaci.2016.02.030 27177780 PMC5014699

[bib61] Noval Rivas, M., O.T. Burton, P. Wise, L.M. Charbonnier, P. Georgiev, H.C. Oettgen, R. Rachid, and T.A. Chatila. 2015. Regulatory T cell reprogramming toward a Th2-cell-like lineage impairs oral tolerance and promotes food allergy. Immunity. 42:512–523. 10.1016/j.immuni.2015.02.004 25769611 PMC4366316

[bib62] Noval Rivas, M., and T.A. Chatila. 2016. Regulatory T cells in allergic diseases. J. Allergy Clin. Immunol. 138:639–652. 10.1016/j.jaci.2016.06.003 27596705 PMC5023156

[bib63] Osterfeld, H., R. Ahrens, R. Strait, F.D. Finkelman, J.C. Renauld, and S.P. Hogan. 2010. Differential roles for the IL-9/IL-9 receptor α-chain pathway in systemic and oral antigen-induced anaphylaxis. J. Allergy Clin. Immunol. 125:469–476.e2. 10.1016/j.jaci.2009.09.054 20159257 PMC4259249

[bib64] Patil, S.U., A.O. Ogunniyi, A. Calatroni, V.R. Tadigotla, B. Ruiter, A. Ma, J. Moon, J.C. Love, and W.G. Shreffler. 2015. Peanut oral immunotherapy transiently expands circulating Ara h 2-specific B cells with a homologous repertoire in unrelated subjects. J. Allergy Clin. Immunol. 136:125–134.e12. 10.1016/j.jaci.2015.03.026 25985925 PMC4494892

[bib65] Patil, S.U., J. Steinbrecher, A. Calatroni, N. Smith, A. Ma, B. Ruiter, Y. Virkud, M. Schneider, and W.G. Shreffler. 2019. Early decrease in basophil sensitivity to Ara h 2 precedes sustained unresponsiveness after peanut oral immunotherapy. J. Allergy Clin. Immunol. 144:1310–1319.e4. 10.1016/j.jaci.2019.07.028 31377342 PMC6905043

[bib66] Prussin, C., J. Lee, and B. Foster. 2009. Eosinophilic gastrointestinal disease and peanut allergy are alternatively associated with IL-5^+^ and IL-5^−^ T_H_2 responses. J. Allergy Clin. Immunol. 124:1326–1332.e6. 10.1016/j.jaci.2009.09.048 20004787 PMC2994258

[bib67] Qamar, N., A.B. Fishbein, K.A. Erickson, M. Cai, C. Szychlinski, P.J. Bryce, R.P. Schleimer, R.L. Fuleihan, and A.M. Singh. 2015. Naturally occurring tolerance acquisition to foods in previously allergic children is characterized by antigen specificity and associated with increased subsets of regulatory T cells. Clin. Exp. Allergy. 45:1663–1672. 10.1111/cea.12570 25989379 PMC4607624

[bib68] Rachid, R., E. Stephen-Victor, and T.A. Chatila. 2021. The microbial origins of food allergy. J. Allergy Clin. Immunol. 147:808–813. 10.1016/j.jaci.2020.12.624 33347905 PMC8096615

[bib69] Ramsey, N., and M.C. Berin. 2021. Pathogenesis of IgE-mediated food allergy and implications for future immunotherapeutics. Pediatr. Allergy Immunol. 32:1416–1425. 10.1111/pai.13501 33715245 PMC9096874

[bib70] Ruiter, B., N.P. Smith, B. Monian, A.A. Tu, E. Fleming, Y.V. Virkud, S.U. Patil, C.A. Whittaker, J.C. Love, and W.G. Shreffler. 2020. Expansion of the CD4^+^ effector T-cell repertoire characterizes peanut-allergic patients with heightened clinical sensitivity. J. Allergy Clin. Immunol. 145:270–282. 10.1016/j.jaci.2019.09.033 31654649 PMC6949413

[bib71] Sampath, V., E.M. Abrams, B. Adlou, C. Akdis, M. Akdis, H.A. Brough, S. Chan, P. Chatchatee, R.S. Chinthrajah, R.R. Cocco, . 2021. Food allergy across the globe. J. Allergy Clin. Immunol. 148:1347–1364. 10.1016/j.jaci.2021.10.018 34872649

[bib72] Sampson, H.A. 2001. Utility of food-specific IgE concentrations in predicting symptomatic food allergy. J. Allergy Clin. Immunol. 107:891–896. 10.1067/mai.2001.114708 11344358

[bib73] Sampson, H.A., D.Y. Leung, A.W. Burks, G. Lack, S.L. Bahna, S.M. Jones, and D.A. Wong. 2011. A phase II, randomized, double-blind, parallel-group, placebo-controlled oral food challenge trial of Xolair (omalizumab) in peanut allergy. J. Allergy Clin. Immunol. 127:1309–1310.e1. 10.1016/j.jaci.2011.01.051 21397314

[bib74] Santos, A.F., L.K. James, H.T. Bahnson, M.H. Shamji, N.C. Couto-Francisco, S. Islam, S. Houghton, A.T. Clark, A. Stephens, V. Turcanu, . 2015. IgG inhibits peanut-induced basophil and mast cell activation in peanut-tolerant children sensitized to peanut major allergens. J. Allergy Clin. Immunol. 135:1249–1256. 10.1016/j.jaci.2015.01.012 25670011 PMC4418748

[bib75] Schneider, L.C., R. Rachid, J. LeBovidge, E. Blood, M. Mittal, and D.T. Umetsu. 2013. A pilot study of omalizumab to facilitate rapid oral desensitization in high-risk peanut-allergic patients. J. Allergy Clin. Immunol. 132:1368–1374. 10.1016/j.jaci.2013.09.046 24176117 PMC4405160

[bib76] Skripak, J.M., S.D. Nash, H. Rowley, N.H. Brereton, S. Oh, R.G. Hamilton, E.C. Matsui, A.W. Burks, and R.A. Wood. 2008. A randomized, double-blind, placebo-controlled study of milk oral immunotherapy for cow’s milk allergy. J. Allergy Clin. Immunol. 122:1154–1160. 10.1016/j.jaci.2008.09.030 18951617 PMC3764488

[bib77] Suárez-Fariñas, M., M. Suprun, H.L. Chang, G. Gimenez, G. Grishina, R. Getts, K. Nadeau, R.A. Wood, and H.A. Sampson. 2019. Predicting development of sustained unresponsiveness to milk oral immunotherapy using epitope-specific antibody binding profiles. J. Allergy Clin. Immunol. 143:1038–1046. 10.1016/j.jaci.2018.10.028 30528770 PMC7658687

[bib78] Suprun, M., G. Grishina, A. Henning, S. Sicherer, R. Wood, S. Jones, W. Burks, D. Leung, M. Suarez-Farinas, and H. Sampson. 2018. Peanut epitope-specific IgE binding can predict clinical peanut allergy. Allergy. 73:116.

[bib79] Suprun, M., P. Kearney, C. Hayward, H. Butler, R. Getts, S.H. Sicherer, P.J. Turner, D.E. Campbell, and H.A. Sampson. 2022a. Predicting probability of tolerating discrete amounts of peanut protein in allergic children using epitope-specific IgE antibody profiling. Allergy. 77:3061–3069. 10.1111/all.15477 35960650 PMC10286745

[bib80] Suprun, M., S.H. Sicherer, R.A. Wood, S.M. Jones, D.Y.M. Leung, A.W. Burks, D. Dunkin, M. Witmer, G. Grishina, R. Getts, . 2022b. Mapping sequential IgE-binding epitopes on major and minor egg allergens. Int. Arch. Allergy Immunol. 183:249–261. 10.1159/000519618 34818647

[bib81] Suprun, M., S.H. Sicherer, R.A. Wood, S.M. Jones, D.Y.M. Leung, A.K. Henning, P. Dawson, A.W. Burks, R. Lindblad, R. Getts, . 2020. Early epitope-specific IgE antibodies are predictive of childhood peanut allergy. J. Allergy Clin. Immunol. 146:1080–1088. 10.1016/j.jaci.2020.08.005 32795587 PMC8095129

[bib82] Syed, A., M.A. Garcia, S.C. Lyu, R. Bucayu, A. Kohli, S. Ishida, J.P. Berglund, M. Tsai, H. Maecker, G. O’Riordan, . 2014. Peanut oral immunotherapy results in increased antigen-induced regulatory T-cell function and hypomethylation of forkhead box protein 3 (FOXP3). J. Allergy Clin. Immunol. 133:500–510. 10.1016/j.jaci.2013.12.1037 24636474 PMC4121175

[bib83] Tang, M.L., A.L. Ponsonby, F. Orsini, D. Tey, M. Robinson, E.L. Su, P. Licciardi, W. Burks, and S. Donath. 2015. Administration of a probiotic with peanut oral immunotherapy: A randomized trial. J. Allergy Clin. Immunol. 135:737–744.e8. 10.1016/j.jaci.2014.11.034 25592987

[bib84] Thyagarajan, A., S.M. Jones, A. Calatroni, L. Pons, M. Kulis, C.S. Woo, M. Kamalakannan, B.P. Vickery, A.M. Scurlock, A. Wesley Burks, and W.G. Shreffler. 2012. Evidence of pathway-specific basophil anergy induced by peanut oral immunotherapy in peanut-allergic children. Clin. Exp. Allergy. 42:1197–1205. 10.1111/j.1365-2222.2012.04028.x 22805467 PMC3779434

[bib85] Tsai, M., K. Mukai, R.S. Chinthrajah, K.C. Nadeau, and S.J. Galli. 2020. Sustained successful peanut oral immunotherapy associated with low basophil activation and peanut-specific IgE. J. Allergy Clin. Immunol. 145:885–896.e6. 10.1016/j.jaci.2019.10.038 31805311 PMC6957313

[bib86] Turcanu, V., S.J. Maleki, and G. Lack. 2003. Characterization of lymphocyte responses to peanuts in normal children, peanut-allergic children, and allergic children who acquired tolerance to peanuts. J. Clin. Invest. 111:1065–1072. 10.1172/JCI200316142 12671056 PMC152580

[bib87] van Wijk, F., S. Hoeks, S. Nierkens, S.J. Koppelman, P. van Kooten, L. Boon, L.M. Knippels, and R. Pieters. 2005. CTLA-4 signaling regulates the intensity of hypersensitivity responses to food antigens, but is not decisive in the induction of sensitization. J. Immunol. 174:174–179. 10.4049/jimmunol.174.1.174 15611239

[bib88] Varshney, P., S.M. Jones, L. Pons, M. Kulis, P.H. Steele, A.R. Kemper, A.M. Scurlock, T.T. Perry, and A.W. Burks. 2009. Oral immunotherapy (OIT) induces clinical tolerance in peanut-allergic children. J. Allergy Clin. Immunol. 123:665. 10.1016/j.jaci.2008.12.65619281911

[bib89] Varshney, P., S.M. Jones, A.M. Scurlock, T.T. Perry, A. Kemper, P. Steele, A. Hiegel, J. Kamilaris, S. Carlisle, X. Yue, . 2011. A randomized controlled study of peanut oral immunotherapy: Clinical desensitization and modulation of the allergic response. J. Allergy Clin. Immunol. 127:654–660. 10.1016/j.jaci.2010.12.1111 21377034 PMC3060783

[bib90] Vickery, B.P., J.P. Berglund, C.M. Burk, J.P. Fine, E.H. Kim, J.I. Kim, C.A. Keet, M. Kulis, K.G. Orgel, R. Guo, . 2017. Early oral immunotherapy in peanut-allergic preschool children is safe and highly effective. J. Allergy Clin. Immunol. 139:173–181.e8. 10.1016/j.jaci.2016.05.027 27522159 PMC5222765

[bib91] Vickery, B.P., A.M. Scurlock, M. Kulis, P.H. Steele, J. Kamilaris, J.P. Berglund, C. Burk, A. Hiegel, S. Carlisle, L. Christie, . 2014. Sustained unresponsiveness to peanut in subjects who have completed peanut oral immunotherapy. J. Allergy Clin. Immunol. 133:468–475. 10.1016/j.jaci.2013.11.007 24361082 PMC3960331

[bib92] Virkud, Y.V., A.W. Burks, P.H. Steele, L.J. Edwards, J.P. Berglund, S.M. Jones, A.M. Scurlock, T.T. Perry, R.D. Pesek, and B.P. Vickery. 2017. Novel baseline predictors of adverse events during oral immunotherapy in children with peanut allergy. J. Allergy Clin. Immunol. 139:882–888.e5. 10.1016/j.jaci.2016.07.030 27609653 PMC5337444

[bib93] Wambre, E., V. Bajzik, J.H. DeLong, K. O’Brien, Q.A. Nguyen, C. Speake, V.H. Gersuk, H.A. DeBerg, E. Whalen, C. Ni, . 2017. A phenotypically and functionally distinct human T_H_2 cell subpopulation is associated with allergic disorders. Sci. Transl. Med. 9:9. 10.1126/scitranslmed.aam9171 PMC5987220 28768806

[bib94] Warren, C.M., J. Jiang, and R.S. Gupta. 2020. Epidemiology and burden of food allergy. Curr. Allergy Asthma Rep. 20:6. 10.1007/s11882-020-0898-7 32067114 PMC7883751

[bib95] Weissler, K.A., M. Rasooly, T. DiMaggio, H. Bolan, D. Cantave, D. Martino, M.R. Neeland, M.L.K. Tang, T.D. Dang, K.J. Allen, . 2018. Identification and analysis of peanut-specific effector T and regulatory T cells in children allergic and tolerant to peanut. J Allergy Clin Immunol. 141:1699–1710 e1697. 10.1016/j.jaci.2018.01.035 29454004 PMC5938104

[bib96] Werfel, T., R. Asero, B.K. Ballmer-Weber, K. Beyer, E. Enrique, A.C. Knulst, A. Mari, A. Muraro, M. Ollert, L.K. Poulsen, . 2015. Position paper of the EAACI: Food allergy due to immunological cross-reactions with common inhalant allergens. Allergy. 70:1079–1090. 10.1111/all.12666 26095197

[bib97] Wisniewski, J.A., S.P. Commins, R. Agrawal, K.E. Hulse, M.D. Yu, J. Cronin, P.W. Heymann, A. Pomes, T.A. Platts-Mills, L. Workman, and J.A. Woodfolk. 2015. Analysis of cytokine production by peanut-reactive T cells identifies residual Th2 effectors in highly allergic children who received peanut oral immunotherapy. Clin. Exp. Allergy. 45:1201–1213. 10.1111/cea.12537 25823600 PMC4472497

[bib98] Wood, R.A., J.S. Kim, R. Lindblad, K. Nadeau, A.K. Henning, P. Dawson, M. Plaut, and H.A. Sampson. 2016. A randomized, double-blind, placebo-controlled study of omalizumab combined with oral immunotherapy for the treatment of cow’s milk allergy. J. Allergy Clin. Immunol. 137:1103–1110.e11. 10.1016/j.jaci.2015.10.005 26581915 PMC5395304

[bib99] Wright, B.L., M. Kulis, K.A. Orgel, A.W. Burks, P. Dawson, A.K. Henning, S.M. Jones, R.A. Wood, S.H. Sicherer, and R.W. Lindblad, . 2016. Component-resolved analysis of IgA, IgE, and IgG4 during egg OIT identifies markers associated with sustained unresponsiveness. Allergy. 71:1552–1560. 10.1111/all.12895 27015954 PMC5035709

[bib100] Xiong, H., J. Dolpady, M. Wabl, M.A. Curotto de Lafaille, and J.J. Lafaille. 2012. Sequential class switching is required for the generation of high affinity IgE antibodies. J. Exp. Med. 209:353–364. 10.1084/jem.20111941 22249450 PMC3280879

[bib101] Zhang, Y., F. Collier, G. Naselli, R. Saffery, M.L. Tang, K.J. Allen, A.L. Ponsonby, L.C. Harrison, P. Vuillermin, and B.I.S.I. Group. 2016. Cord blood monocyte-derived inflammatory cytokines suppress IL-2 and induce nonclassic “TH2-type” immunity associated with development of food allergy. Sci. Transl. Med. 8:321ra328. 10.1126/scitranslmed.aad4322 26764159

